# Impact of different levels of probiotic on productive performance, nutrient retention of broiler chickens fed low protein diets

**DOI:** 10.5455/javar.2023.j692

**Published:** 2023-09-24

**Authors:** Zeyad Kamal Imari, Hayder Raheem Alnajm, Sarah Jasim Zamil

**Affiliations:** Department of Animal Production Techniques, Technical College of Al-Musaib, Al-Furat Al-Awsat Technical University, AL-Kufa, Iraq

**Keywords:** Protein reduction, broiler, probiotic, growth performance

## Abstract

**Objective:**

This research assesses whether probiotics could enhance growth performance and improve nutrient digestibility in birds fed with low-protein diets.

**Materials and Methods:**

A total of 250 1-day-old ROSS chicks were used in a completely randomized design consisting of 5 treatments with 5 replicates and 10 birds for each replicate. The experimental diets were as follows: basal diet containing 100% crude protein (CP) according to Nutrition Research Council recommendation (control diet); basal diet containing CP 10% less than the control diet low protein (LP); LP with addition of probiotic by 50 mg/kg diet (LP + P1); LP with addition of probiotics by 100 mg/kg diet (LP + P2); LP with an addition of probiotics by 150 mg/kg diet (LP + P3).

**Results:**

Broilers fed with the control, LP + P2, and LP + P3 diets had greater body weight and weight gain than broilers fed with the LP during starter, finisher, and total periods (*p < *0.01). The feed conversion ratio was the best (*p < *0.01) in the control and LP + P3 treatments compared with the LP and LP + P1 treatments throughout the experiment. The European production efficiency factor was the lowest in the LP treatment compared with other treatments from 1 to 42 days. Protein efficiency ratio and protein retention were both higher in birds fed with the LP + P2 and LP + P3 diets compared to birds given the control and LP diets (*p *< 0.01*).*

**Conclusion:**

Adding probiotics to the diet remarkably improved the productive performance and nutrient digestibility of broiler-fed low-protein diets.

## Introduction

Environmental pollution is one of the important problems that directly or indirectly affect human and animal health. The poultry industry may contribute to increasing environmental and health problems [1]. Poultry manure contains high levels of organic and inorganic materials [2]. The excreted nitrogen in the animal manure contributes to atmospheric pollution through ammonia emissions and water pollution through the eutrophication phenomenon [3]. Therefore, some studies have proposed to reduce nutrient excretions and feed costs by minimizing the quantity of the provided feed [4]. Or through restricting the quality of feed by reducing protein percentage [5], reducing metabolizable energy [6], or both at the same time [7]. However, using feed-restricted programs may reduce the growth performance (GP) of broiler chickens. Ding et al. [8] reported that the body weight (BW), weight gain (WG), and fed conversion ratio were significantly impaired when the dietary protein was reduced from 21% to 19% throughout the starter phase and from 19% to 17% during the grower phase.

Reducing crude protein (CP) by 15% of the Nutrition Research Council (NRC) recommended level significantly impairs the productive performance of birds at periods 1–21, 22–42, and 1–42 days of age [9]. Hu et al. [10] observed that the feed intake (FI) had increased, and the feed conversion ratio (FCR) had deteriorated when the energy diet was reduced from 3,200 to 2,900 kcal/kg. Lowering energy and protein in the diet significantly decreases BW, egg weight, and albumen ratio [11]. Therefore, some studies have resorted to using feed additives to reduce the negative effects of low protein (LP) or low energy concentrations in the diet. One of these additives is probiotics, which are nonpathogenic microorganisms that have a positive effect on the host by increasing the proliferation of beneficial bacteria and decreasing the generation of pathogenic bacteria [12]. Stimulation of the immune response [13], improving nutrient digestibility, energy metabolism, and GP, as well as reducing feed costs [14]. Thus, this study set out to assess the role of probiotics in mitigating the detrimental effects of a lower protein diet on the productive performance of broiler chickens by enhancing nutrient digestibility, decreasing nitrogen excretion, and evaluating the effect of reducing dietary protein on improving the digestibility of nutrients and reducing the amount of excreted nitrogen.

## Materials and Methods 

### Ethical approval

The study was performed on the poultry farm at the College of Technical Al-Musaib of Alfurat Alawsat Technical University, and ethical approval was obtained from the institutional ethics committee of Alfurat Alawsat Technical University to conduct the animal experiment [Animal Care and Experiment Ethics Committee No. 1016 on 5/9/2022].

### Birds, diet, and housing 

A total of 250 1-day-old ROSS^308^ broiler chicks were purchased from a local hatchery and their average live BW was 43.02 ± 1.08 gm. Using a complete design, we randomly divided the birds into 5 treatments with 5 replicates for each treatment and 10 chicks for each replicate. The treatments were as follows: basal diet without reduction or additional control; basal diet with reduction of protein level by 10% less than NRC’s recommendation without probiotic addition (LP); LP with probiotic at 50 mg/kg diet (LP + P1); LP with probiotic at 100 mg/kg diet (LP + P2), and LP with probiotic at 150 mg/kg diet (LP + P3). All birds were randomly housed in 25-floor pens 1 × 1.25 m^2^. The temperature of the birds’ house was adjusted to 32°C during the first 7 days. After that, the temperature declined by 2°C weekly to reach 22°C at the end of the experiment. Chicks were supplied with 24 h of light in the first week, followed by a decrease of 1 h of light per week until they reached 19 h of light in the sixth week. Except for protein, the nutritional requirements of broilers were supplied by the recommendations of the NRC (Table 1). All birds have free access to food and water.

### Growth performance 

The BW, weight of provided feed, and weight of residual feed per pen were measured to calculate WG, FI, and FCR. Birds’ weight and the number of days they lived before death were recorded to calculate hen-day (HD) using the following formulas:


$$\mathrm{Daily}\;\mathrm{average}\;\mathrm{WG}=\frac{\mathrm{ABW}2-\mathrm{ABW}1}{\mathrm{number}\;\mathrm{of}\;\mathrm{days}\;\mathrm{for}\;\mathrm{each}\;\mathrm{period}}$$

where BW^1^ refers to the live BW of birds at the beginning period, while BW^2^ refers to the live BW of birds at the end period.

HD = (number of birds at the end stage per replicate× stage in days) + number of days that the birds spent before death


$$\mathrm{Daily}\;\mathrm{average}\;\mathrm{FI}=\frac{\mathrm{total}\;\mathrm{FI}\;\mathrm{per}\;\mathrm{pen}\;\mathrm{in}\;\mathrm{each}\;\mathrm{period}}{\mathrm{HD}}$$

$$\mathrm{FCR}=\frac{\mathrm{total}\;\mathrm{FI}\;\mathrm{per}\;\mathrm{pen}\;\mathrm{in}\;\mathrm{each}\;\mathrm{period}}{(\mathrm{ABW}2-\mathrm{ABW}1)+\mathrm{weight}\;\mathrm{of}\;\mathrm{dead}\;\mathrm{birds}}.$$

### European production efficiency factor (EPEF) and protein efficiency ratio (PER)

The EPEF and PER were computed based on the description that was pointed out by Lukić et al. [15] and Kamran et al. [16], respectively,


$$\mathrm{EPEF}=\frac{\mathrm{Livability}\;\times \;\mathrm{ABW}(\mathrm{kg})}{\mathrm{FCR}\;\times \;\mathrm{birdage}\;(\mathrm{day})}\times 100$$

$$\mathrm{Livability}=\frac{\mathrm{number}\;\mathrm{of}\;\mathrm{birds}\;\mathrm{at}\;\mathrm{the}\;\mathrm{end}\;\mathrm{each}\;\mathrm{of}\;\mathrm{period}}{\mathrm{number}\;\mathrm{of}\;\mathrm{birds}\;\mathrm{at}\;\mathrm{the}\;\mathrm{beginning}\;\mathrm{of}\;\mathrm{each}\;\mathrm{period}}\times 100$$

PER = (WG gm)/(protein intake gm).

### Dry matter (DM), organic matter (OM), CP, and ash retention

The total collection method of excreta was to estimate the retention of DM, CP, ether extract (EE), OM, and ash. The nutrient retention trial started on the 42nd day of age (at the end of the original trial). One bird per replicate was selected, and the selected birds were placed in individual metabolic cages. The retention trial consisted of a 2-day adaptation period and a 3-day collection. Five experimental diets were prepared, weighed, and provided to the birds. To mark the experimental diet, 1% chromium oxide was used [17]. The excreta collection period began when the green color appeared and ended when the green color disappeared. The excreta were collected from each cage and weighed daily. After that, the excreta from each cage was combined and stored in the freezer at −20°C until processed later. The FI was also measured during the collection period.

**Table 1.** Feeds and nutritional composition of experimental rations during the initial phase (11–24 days) and finisher phase (25–42 days).

Treatments	Experimental diets (11–24 days)					Experimental diets (25–42 days)				
	C	LP	LP + P1	LP + P2	LP + P3	C	LP	LP + P1	LP + P2	LP + P3
**CP level %**	**23**	**20.7**	**20.7**	**20.7**	**20.7**	**20**	**18**	**18**	**18**	**18**
**Probiotic mg/kg**	**0**	**0**	**50**	**100**	**150**	**0**	**0**	**50**	**100**	**150**
Ingredients %										
Corn	45.9	54.11	54.11	54.11	54.11	55.35	62.50	62.50	62.50	62.50
SBM (44% CP)	42.65	35.72	35.72	35.72	35.72	34.27	28.14	28.14	28.14	28.14
Corn oil	6.75	5.37	5.37	5.37	5.37	6.50	5.28	5.28	5.28	5.28
DCP	1.75	1.83	1.83	1.83	1.83	1.25	1.32	1.32	1.32	1.32
LS	1.3	1.31	1.31	1.31	1.31	1.37	1.38	1.38	1.38	1.38
Table salt	0.27	0.27	0.27	0.27	0.27	0.18	0.18	0.18	0.18	0.18
NaHCO_3_	0.45	0.44	0.44	0.44	0.44	0.34	0.34	0.34	0.34	0.34
VP^1^	0.26	0.26	0.2	0.26	0.26	0.26	0.26	0.26	0.26	0.26
TMP	0.24	0.24	0.24	0.24	0.24	0.24	0.24	0.24	0.24	0.24
DL-Met	0.18	0.24	0.24	0.24	0.24	0.08	0.14	0.14	0.14	0.14
L-Lys	0.19	0.19	0.19	0.19	0.19	0.14	0.14	0.14	0.14	0.14
L-Thre	0.06	0.06	0.06	0.06	0.06	0.02	0.08	0.08	0.08	0.08
Total	99.99	100.03	99.98	100.04	100.04	100	100	100	100	100
Calculated nutrients										
ME, kcal/kg	3,100	3,100	3,100	3,100	3,100	3,200	3,200	3,200	3,200	3,200
CP%	23	20.7	20.7	20.7	20.7	20	18	18	18	18
CF%	3.98	3.68	3.68	3.68	3.68	3.60	3.35	3.35	3.35	3.35
Ca%	1.0	1.0	1.0	1.0	1.0	0.9	0.9	0.9	0.9	0.9
P%	0.45	0.45	0.45	0.45	0.45	0.35	0.35	0.35	0.35	0.35
Na%	0.24	0.24	0.24	0.24	0.24	0.18	0.18	0.18	0.18	0.18
Lys%	1.4	1.23	1.23	1.23	1.23	1.16	1.02	1.02	1.02	1.02
Met%	0.55	0.58	0.58	0.58	0.58	0.40	0.43	0.43	0.43	0.43
Met + Cysteine%	0.9	0.9	0.9	0.9	0.9	0.72	0.72	0.72	0.72	0.72
Threonine%	0.86	0.8	0.8	0.8	0.8	0.74	0.74	0.74	0.74	0.74
Arginine	1.56	1.39	1.39	1.39	1.39	1.35	1.19	1.19	1.19	1.19
Tryptophan	0.22	0.20	0.20	0.20	0.20	0.19	0.18	0.18	0.18	0.18

^1^Provided per kg of diet: Retinol, 7,000 U; vit. D3, 2,200 U; vit. E,18U; vit. K3, 2.5 mg; vit. B1,1.7 mg; vit. B1, 1.5 mg; vit. B2, 6.7 mg; vit. B6, 3 mg; vit. B12, 0.04 mg; vit. B6, 0.16 mg; vit. B5, 29 mg; vit. B3, 11 mg; ChCl, 1,100 mg; vit.C, 340 mg; and vit. B9, 1.1 mg.

Ca, calcium; P, phosphorus; DCP, Dicalcium-Phosphate; VP, vitamin premix; TMP, trace mineral premix; CP, crude protein; LS, Limestone; DL-Met: DL-Methionine; L-Lys: L-lysine-HCl; L-Thr: L-threonine.

### Chemical analysis 

The excreta and dried diet specimens were crushed and prepared for subsequent chemical assays. Diet and excreta specimens were analyzed for DM by the drying method and CP by the Kjeldahl method referred to by Gallardo et al. [18]. The diets and excreta specimens were ashed by a muffle furnace, as described by Sadeghi [19].

### Statistical analysis

For this study, we analyzed the effect of probiotics on the GP attributes of birds fed with low-protein diets using a one-way ANOVA performed in the SAS 9.4 program. Duncan’s multiple range test was utilized to examine statistically significant variations in treatment averages at the level of (*p < *0.05).

## Results and Discussion 

### Growth performance 

Table 2 displays the impact of protein reduction and probiotic addition on BW, WG, FI, and FCR of the broiler. The live BW and BW gain significantly increased (*p* < 0.01) in birds receiving basal diet (control diet) and the birds fed with LP diet plus probiotic by 150 mg/kg diet (LP + P3) compared with birds fed with LP diet without probiotic (LP) or those receiving LP plus probiotic by 50 mg/kg diet (LP + P1) from 1 to 21 days. In the same period, a significant superiority in BW and WG was observed in the LP + P2 treatment compared to the LP treatment. The treatments did not affect FI (*p *> 0.05) throughout the starter, finisher, and total periods. In starter periods, the best-fed conversion ratio was observed in the control treatment, followed by the LP + P3 treatment, compared with other groups (*p *= 0.036). During the finisher and total phases, the FCR was lowest in the LP treatment compared to other treatments (*p < *0.01).

Our results concord with the finding by Tajudeen et al. [20] that birds’ ability to grow during the starter and finisher stages is negatively affected by a 1% decrease in dietary protein at the standard level. Reducing dietary protein from 20% to 16% decreases the WG of the body and the FI of layer chicks from 7 to 56 days [21]. Compared to birds fed a standard protein diet or birds fed with a high protein diet, Barekatain et al. [22] observed that reducing dietary protein impairs broilers’ productivity. The results of the current research demonstrated that probiotic supplementation improved the growth of birds fed with low-protein diets. A similar result by Ferdous et al. [23] confirmed that adding probiotics to the diet had improved the BW, WG, and FCR. Adding probiotics to the diet enhances the bird’s productivity [24]. The role of probiotics in boosting the availability of amino acids and minerals or their positive effects on digestive system health may be responsible for improving the growth of birds fed with low dietary protein. According to Poberezhets et al. [25], the inclusion of probiotics in the diet increases the absorption of essential amino acids and minerals, which subsequently increases BW and carcass weight. Probiotics produce bioactive substances such as enzymes, amino acids, volatile fatty acids, vitamins, and bacteriocins, which play important roles in the animal’s body [26]. Probiotic inclusion enhances the absorption of methionine, histidine, valine, leucine, isoleucine, and tyrosine, especially when feeding on plant protein [27]. Adding probiotics to the diet improved GP, the relative weight of carcass parts, gut morphology, and humoral and cellular immune responses [28]. Lei et al. [29] referred to the fact that the addition of probiotics at 60 mg/kg in the diet significantly increases villi height b (VH) and VH to crypt depth ratio (CD ratio) in small intestinal chicks. An increase in VH in small intestinal cells increases surface area for absorption, resulting in improved nutrient absorption and GP [30].

**Table 2.** Effect of different levels of probiotics on GP of broilers fed with LP diets.

Item	Experimental diets					SEM	*p*-value
Starter period (1–21 days)	Control	LP	LP + P1	LP + P2	LP + P3		
BW, gm/bird	848.7^a^	715.7^c^	734.5^bc^	804.1^ab^	849.6^a^	10.62	0.001
WG, gm/bird	805.5^a^	672.6^c^	692.1^bc^	761.3^ab^	806^a^	10.59	0.001
FI, gm/bird	1,110.1	1,030.8	1,038.4	1,082.9	1,119.6	13.66	0.115
FCR, gm/gm	1.38^b^	1.51^a^	1.49^a^	1.42^ab^	1.39^b^	0.015	0.036
Finisher period (21–42)							
BW, gm/bird	2,855.4^a^	2,439.5^c^	2,628.3^b^	2,797.1^a^	2,785.1^ab^	25.18	<0.0001
WG, gm/bird	2,006.7^a^	1,685.7^b^	1,893.8^a^	1,993^a^	1,935.5^a^	19.95	<0.0001
FI, gm/bird	3,202.2	2,958.3	3,147.8	3,184.1	3,118.9	28.15	0.086
FCR, gm/gm	1.59^b^	1.77^a^	1.67^b^	1.61^b^	1.61^b^	0.012	0.002
Total period (1–42)							
WG, gm/bird	2,812.2^a^	2,343.4^c^	2,584^b^	2,753.4^ab^	2,741.5^ab^	25.86	<0.0001
FI, gm/bird	4,312.4	3,980	4,208.2	4,302.8	4,238.5	39.59	0.092
FCR, gm/gm	1.53^c^	1.70^a^	1.62^b^	1.56^bc^	1.54^c^	0.009	<0.0001

^a,b,c^ Values in the same row with different letters have dramatically different values (*p <* 0.05).

Control diet: it is the basal diet with a standard level of CP according to the Nutrition Research Council (NRC); LP: basal diet containing CP 10% less than the control diet without the addition of probiotic; LP + P1, LP + P2, and LP + P3: basal diet containing CP 10% less than control diet with the addition of probiotics by 50, 100, and 150 mg/kg diet, respectively; SEM: standard error of the mean.

**Table 3.** Effect of different levels of probiotics on EPEF, protein intake, and PER of broilers fed with LP diets.

Item	Experimental diets					SEM	*p*-value
Starter period (1–21 days)	Control	LP	LP + P1	LP + P2	LP + P3		
Mortality rate %	0.0	4.0	2.0	0.0	0.0	0.632	0.213
EPEF	279.2^a^	204.2^b^	215.72^b^	255.4^a^	277^a^	5.89	0.001
Protein intake	255.33^a^	210.56^c^	214.95^bc^	224.15^bc^	231.77^b^	2.924	0.001
PER gm/gm	3.15^b^	3.20^b^	3.22^b^	3.39^ab^	3.47^a^	0.036	0.048
Finisher period (21–42)							
Mortality rate %	0.0	2.0	2.0	2.0	0.0	0.692	0.736
EPEF	599.3^a^	446.1^c^	526.9^b^	577.1^ab^	572.3^ab^	8.49	<0.0001
Protein intake	640.4^a^	532.5^c^	566.6^bc^	573.13^b^	561.4^bc^	5.17	<0.0001
PER gm/gm	3.13^c^	3.16^bc^	3.34^ab^	3.47^a^	3.44^a^	0.028	0.002
Total period (1–42)							
Mortality rate%	0.0	6.0	4.0	2.0	0.0	0.80	0.115
EPEF	437.1^a^	309^c^	362.38^b^	412.4^a^	422.25^a^	7.80	<0.0001
Protein intake	927.2^a^	770.2^b^	814.29^b^	832.59^b^	820.2^b^	7.64	<0.0001
PER gm/gm	3.03^c^	3.04^c^	3.17^bc^	3.30^ab^	3.34^a^	0.022	<0.0001

^a,b,c^ Values in the same row with different letters have dramatically different values (*p <* 0.05).

Control diet: it is the basal diet with a standard level of CP according to the Nutrition Research Council (NRC). LP: a basal diet containing CP 10% less than the control diet without the addition of probiotic; LP + P1, LP + P2, and LP + P3: a basal diet containing CP 10% less than the control diet with the addition of probiotic by 50, 100, and 150 mg/kg diet, respectively; SEM: standard error of the mean; EPEF: European production efficiency factor.

### Mortality rate, EPEF, protein intake, and PER

Table 3 exhibits the mortality rate, EPEF, protein intake, and PER. The EPEF was higher in the control, LP + P2, and LP + P3 treatments compared to the LP treatment during the starter stage (*p < *0.01), finisher stage (*p < *0.001), and total stage (*p < *0.001). At the starter, finisher, and whole stages, chickens fed with the control diet had the highest protein intake relative to the other groups (*p < *0.01, *p < *0.001, and *p < *0.001, respectively). The PER was higher in birds fed with the LP + P3 diet than in other birds, except those fed the LP + P2 diet during 1–21 days. The same trend was observed in the finisher period; birds given the LP + P2 diet, as well as birds fed with the LP + P3 diet, had higher PER (*p = *0.002) compared to birds receiving the LP diet or chicks fed with the control diet. In the total period, the PER was significantly increased (*p < *0*.*0001) in the LP + P3 and LP + P2 treatments compared to the control and LP treatments.

The results of the current study were compatible with those of Van Harn et al. [31] who observed that lowering dietary protein from 208 to 178 gm/kg at the grower period and decreasing dietary protein from 198 to 168 gm/kg at the finisher period did not have an effect on mortality rate. The European performance efficiency factor significantly decreased when the CP in the diet decreased [32]. Van Harn et al. [31] indicated that reducing dietary protein raises the PER. Since there is a positive correlation between the GP and EPEF, the EPEF may decrease when the GP decreases. Law et al. [33] found that during the starter and finisher phases, birds fed with a low-protein diet had lower BW, weight increase, feed consumption, and carcass features than birds fed with a standard-protein diet. Reducing CP from 19.3 to 18.8 leads to impaired GP in broiler chickens at 35 days [34]. The enhancement in the EPEF and PER is due to the positive effects of probiotics on the availability, digestion, and absorption of nutrients, which positively reflect on birds’ performance. Bogucka et al. [35] found that the addition of probiotics to the diet enhanced intestinal microbiota balance, gut morphology, and digestive enzymes, which in turn improved nutritional digestion and absorption. Probiotics contribute to improving the protein, fat, and nitrogen-free extract digestibility of broiler chickens [36]. Compared to broilers fed with a control diet, broilers fed with a probiotic-supplemented diet exhibited a higher value of nutrient digestibility [37]. Reis et al. [38] suggested that adding probiotics to broiler chickens’ diets increases the digestibility of metabolizable energy, DM, and crud protein.

### Nutrients retention

The results indicated that, compared to the other treatments, the control treatment had considerably lower (*p < *0.01) retention of DM and OM (Table 4). Birds fed with the LP + P1, LP + P2, and LP + P3 diets have higher ash retention (*p* = 0.008) when compared with birds receiving the LP diet or chicks fed with the control diet. The protein retention was higher (*p = *0.001) in the LP + P2 and LP + P3 treatments compared to the LP and control treatments. There was no remarkable difference (*p < *0.05) between the control treatment and the LP treatment on ash and protein retention. These results agreed with Chrystal et al. [39], who suggested that reducing protein in diets enhances the digestibility of nitrogen and amino acids. The pH content and nitrogen excretion decreased when the dietary protein was reduced from 21.9% to 18% [40]. In addition, reducing dietary protein from 21.5% to 19% increases the digestibility of OM, CP, and EE and decreases protein excretion [41].

**Table 4.** Effect of different levels of probiotics on some of nutrients retention of broilers fed with LP diets.

Apparent total tract retention%	Experimental diets					SEM	*p*-value
	Control	LP	LP + P1	LP + P2	LP + P3		
DM	56.22^b^	62.94^a^	65.34^a^	67.18^a^	66.92^a^	0.761	0.001
OM	58.37^b^	65.1^a^	67.08^a^	68.88^a^	68.50^a^	0.805	0.003
Ash	27.89^b^	30.01^b^	38.56^a^	39.88^a^	40.16^a^	1.221	0.008
Protein	62.53^c^	65.27^bc^	68.79^ab^	70.15^a^	69.94^a^	0.547	0.001

^a,b,c^ Values with different letters within the same row are significantly different (*p <* 0.05).

Control diet: it is the basal diet with a standard level of CP according to the Nutrition Research Council (NRC); LP: basal diet containing CP 10% less than the control diet without the addition of probiotic; LP + P1, LP + P2, and LP + P3: basal diet containing CP 10% less than control diet with addition of probiotic by 50, 100, and 150 mg/kg diet, respectively; SEM: standard error of the mean.

Our data demonstrated that the addition of probiotics to the diets was important in improving nutrient digestibility. Similarly, Zhang et al. [42] found that probiotic supplementation increases amino acid digestibility in the ileum and lowers ammonia concentration. Birds fed with a probiotic-containing feed at 2 gm/kg had significantly higher digestibility of DM, CP, EE, and crude fiber than birds fed with the basal diet without probiotics [43]. Recently, Yang et al. [44] reported that adding probiotics to the diet increases the digestibility of protein, gross energy, and DM. The improvement in digestibility in birds fed with a diet containing probiotics may be attributed to the efficiency of probiotics in increasing beneficial microorganism counts and reducing pathogenic bacteria counts, as well as their role in activating and producing enzymes and improving gut morphology. Zhang et al. [42] noticed that the number of useful bacteria was considerably higher (*p < *0.05), and the number of harmful bacteria was considerably lower (*p < *0.05) in the ceca of birds fed with probiotics when compared with birds fed with a control diet. Another study confirmed that the addition of probiotics led to an inhibition of the numbers of *Clostridium* and an increase of *Bifidobacteria* species in the cecum [45]. Compared to the control treatment, birds fed with probiotics had a maximum VH/CD ratio, villus width, and number of goblet cells [46]. Additionally, Zhang et al. [47] observed that the activities of protease, amylase, and lipase were significantly increased in birds treated with probiotics when compared with birds that were not treated with probiotics.

## Conclusion 

Lowering CP by 10% below the recommended level had a negative impact on chicken growth. Still, probiotic administration at concentrations of 100 and 150 mg/kg diet had a positive influence on productive performance, protein efficiency, and nutrient digestibility.

## List of Abbreviations

CP, crude protein; DM, dry matter; EE, ether extract; EPEF, European production efficiency factor; FCR, feed conversion ratio; FI, feed intake; LP, low protein; NRC, Nutrition Research Council; OM, organic matter; PER, protein efficiency ratio.
